# Functional DNA quantification guides accurate next-generation sequencing mutation detection in formalin-fixed, paraffin-embedded tumor biopsies

**DOI:** 10.1186/gm481

**Published:** 2013-08-30

**Authors:** Sachin Sah, Liangjing Chen, Jeffrey Houghton, Jon Kemppainen, Adam C Marko, Robert Zeigler, Gary J Latham

**Affiliations:** 1 Asuragen, Inc, Austin, TX 78744, USA

## Abstract

The formalin-fixed, paraffin-embedded (FFPE) biopsy is a challenging sample for molecular assays such as targeted next-generation sequencing (NGS). We compared three methods for FFPE DNA quantification, including a novel PCR assay (‘QFI-PCR’) that measures the absolute copy number of amplifiable DNA, across 165 residual clinical specimens. The results reveal the limitations of commonly used approaches, and demonstrate the value of an integrated workflow using QFI-PCR to improve the accuracy of NGS mutation detection and guide changes in input that can rescue low quality FFPE DNA. These findings address a growing need for improved quality measures in NGS-based patient testing.

## Background

Approximately 120 years ago, formaldehyde was identified as a superior fixation agent to preserve tissue samples [1]. Since that time, preservation of tissues with formalin-fixed paraffin-embedding (FFPE) procedures has emerged as the method of choice for histological study and archival storage of clinical specimens. More than 400 million FFPE samples are thought to exist and many have clinical annotations such as primary diagnosis, therapeutic regimen, drug response, and recurrence status. These archives represent an invaluable repository of retrospective patient clinical data. Powerful new genomic technologies, such as next-generation sequencing (NGS), promise to unlock the molecular features of such samples and inform the linkage of genotype and phenotype. Achievement of this goal requires that the unintended consequences of the fixation and embedding process and the duration and conditions of storage on nucleic acid quality be accommodated by the profiling methodology to ensure reliable, accurate, and sensitive biomarker detection.

Unfortunately, the FFPE process causes fragmentation and chemical modifications in DNA, such as cross-linking, deamination and adducts [2-4]. These modifications reduce the number of DNA templates available for amplification, and pose significant challenges to efficient PCR. Factors such as type of fixative, fixation time, age and storage conditions of the FFPE block can contribute to problems in diagnostic testing [5]. In light of these challenges, pre-analytical methods such as end-point PCR using different reference gene amplicon lengths have been previously described to help qualify samples for molecular methods downstream [6-8].

Two common methods for DNA quantification are spectrophotometry and fluorometry using DNA-binding dyes. Spectrophotometry offers a simple and nimble way to accurately measure the bulk concentration of high quality DNA and instruments such as the NanoDrop Spectophotometer are readily available. Yet spectrophotometry cannot gauge the molecular damage and fragmentation caused by fixation, embedding, and/or long-term storage, nor can it anticipate the effects on PCR. For example, a recent study comparing the accuracy of different methods to quantify DNA following controlled degradation demonstrated up to a three-fold difference between spectrophotometry and quantitative PCR (qPCR) for DNA fragments of 150 bp; DNA quantification by PCR, but not spectrophotometry, was affected by the extent of DNA shearing [9]. Spectrophotometry is also susceptible to erroneous measurements from contaminants like RNA and other organic solvents, such as phenol, used in DNA extraction. Fluorometric DNA quantification methods, such as the Qubit® assay, offer high analytical sensitivity, high throughput, and improved tolerance to contaminants. As such, an increasing number of laboratories have adopted this approach as a best practice for NGS applications [10].

Limitations in the availability of many clinical specimens drive the need for low DNA inputs into molecular assays. Cutting edge technologies such as NGS can push the boundaries of input DNA material required for in-depth molecular profiling, particularly in cancer [11-14]. FFPE tumor DNA presents a dual challenge for mutation testing, namely requirements for low template input quantities combined with template damage from the fixation and embedding process that resist amplification by PCR. In addition, low quality FFPE DNA can trigger allele dropouts and produce inaccurate results [6,15]. As a result, metrics based on sensitive and quantitative pre-analytical sample characterization are needed to assess the fraction of template molecules that are competent for PCR amplification.

The goal of this study was to establish and integrate quantitative, pre-analytical molecular quality assessments of FFPE DNA with targeted NGS data to ensure accurate and reliable data interpretations. The approach that we describe, which includes the use of an optimized qPCR assay termed quantitative functional index (QFI)-PCR, leverages broadly available instrumentation (that is, a real-time thermal cycler) and quantifies the absolute number of amplifiable templates in a FFPE DNA sample. Importantly, the strategy is designed to quantify amplicons that are of a similar size as those in the target PCR enrichment library to tightly link functional copy numbers determined in the pre-analytical phase to the performance characteristics of amplicon libraries generated in the ensuing analytical phase. Our findings underscore the influence of FFPE DNA quality on NGS results and interpretations and prescribe sample-specific, data-driven metrics that can accommodate the analysis of low quality DNA in diagnostic cancer applications. This guidance is timely given the rapid migration of NGS into clinical test settings.

## Methods

### Study design

**Figure 1:**
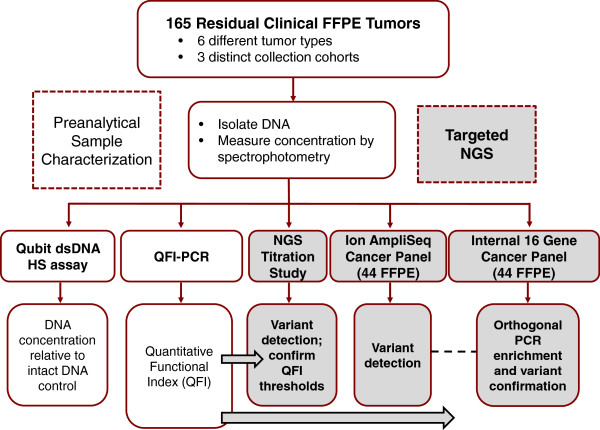
**Study design.** The study design coupled pre-analytical FFPE DNA characterization across three methods (spectrophotometry, fluorescence dye-based quantification, and QFI-PCR) with variant calling results from targeted NGS and confirmation assays to assess the impact of template quality and set thresholds for minimum ‘functional’ DNA inputs. dsDNA, double-stranded DNA; HS, high sensitivity.

The study was designed first to benchmark different methods for pre-analytical FFPE sample characterization and then to synthesize the most informative analyses with the results of targeted NGS-based variant calling (Figure 1). In the initial phase of the study, FFPE DNA assessments determined by spectrophotometry, a sensitive and commonly used fluorescent dye assay (Qubit), and a novel qPCR assay (QFI-PCR) were compared across 165 residual clinical FFPE samples. Quantitative measures of DNA template quality determined by QFI-PCR were then compared with targeted NGS results using defined metrics across varying ‘functional’ FFPE DNA copy numbers to demonstrate DNA quality and input thresholds that supported accurate variant calling. The predictive value of QFI-PCR-based thresholds was established in a titration study, and then applied to a set of 44 FFPE DNA samples interrogated with the Ion AmpliSeq™ Cancer Panel, a commercially available, highly multiplexed PCR method that enriches loci in 46 cancer genes for NGS on the Ion Torrent Personal Genome Machine (PGM; Life Technologies, Carlsbad, CA, USA).

### FFPE specimens and DNA controls

The 165 specimens used in this study were obtained as FFPE blocks from three different sources. Fifty samples that had >70% tumor content and <20% necrosis were selected from Asuragen’s FFPE tumor database. This set included 10 samples each from colon, lung, skin, ovary, and breast tumor biopsies. Seventy-six FFPE blocks containing thyroid tumor biopsies were purchased from Asterand (Detroit, MI, USA). The remaining 39 FFPE tissue specimens were processed from colorectal cancer resections purchased from Folio Biosciences (Columbus, OH, USA) [14]. The ages of the FFPE blocks across all three cohorts ranged from 1 to 18 years. The mean DNA functional quality (QFI; see below) for the Asuragen, Asterand and Folio Biosciences cohorts was 8.0 (95% confidence interval (CI) 6.3 to 9.7), 6.0 (95% CI 4.3 to 7.7) and 3.5 (95% CI 2.1 to 4.9), respectively. No significant difference in the QFI was observed as a function of the age of the FFPE block. All samples were residual de-identified samples acquired in accordance with appropriate human subjects’ regulations using an institutional review board-approved protocol. In addition, Asuragen has filed a Federalwide Assurance for the Protection of Human Subjects (FWA) with the US Department of Health and Human Services.

DNA for all samples was isolated using a modification of the RecoverAll™ Total Nucleic Acid Isolation Kit for FFPE (Life Technologies) [14]. Poor quality samples produced a similar DNA yield and quality following re-extraction. Cell line NA04025 DNA was used as the DNA calibrator for qPCR (NIGMS Human Genetic Cell Repository, Coriell Institute, Camden, NJ, USA).

### DNA copy number quantification using QFI-PCR

DNA samples were initially quantified using NanoDrop spectrophotometer (ThermoScientific, Wilmington, DE, USA) and normalized to 10 ng/μl in deionized water. Samples were further diluted to 5 ng/μl and assessed using qPCR on a 7900HT Fast Real-Time PCR System (Life Technologies). Quantification of amplifiable DNA was assessed by amplifying a 119 bp region in the TATA box binding protein gene (*TBP*). The amplicon length was specifically designed to represent the 120 bp average amplicon size generated for the AmpliSeq™ Cancer Panel. qPCR was carried out in 11 μl reactions with 1× Taqman® Gene Expression Master Mix (Life Technologies), 900 nM forward primer (5′-CCA GAC TGG CAG CAA GAA AAT-3′; Integrated DNA Technologies, Coralville, IA, USA), 900 nM reverse primer (5′-CCT TAT AGG AAA CTT CAC ATC ACA GC-3′; Integrated DNA Technologies), 250 nM Taqman® probe (5′-VIC-TGC TAG AGT TGT ACA GAA GTT GGG TTT TCC AGC-TAMRA-3′; Life Technologies) and 5 ng of DNA. A second qPCR assay (900 nM forward primer 5′-CCT CTG CCT CCG GCA TTT-3′, 900 nM reverse primer 5′-GCC CCC AAG GTT TGC TAT TC-3′, 250 nM probe 5′ 6-FAM/ZEN/3′IB®FQ-CCA GCG TTT TTT GCT TAG GTA TCC AGC TCC-3′; Integrated DNA Technologies), was designed on the ferritin, heavy polypeptide 1 (*FTH1*) gene and also targeted a 119 bp region. The PCR cycling conditions were 95°C for 10 minutes, 50 cycles of 95°C for 15 s, and 60°C for 1 minute. A calibration curve was generated using data from PCR amplification of high quality genomic DNA extracted from the cell line NA04025 (Coriell Cell Repositories, Camden, NJ, USA) using a 5-fold titration series, from 50 ng to 16 pg (15,150 to 5 copies). Copy number - a measure of PCR competency and template ‘functionality’ - was then calculated from the calibration curve. We note that it is critically important to enlist a high quality DNA as a standard to generate the calibration curve since this DNA serves as a reference for a ‘100% functional’ template.

In this study, ‘functionality’ is synonymous with the QFI and is defined as the fraction of haploid DNA templates available for amplification compared to the calibrator DNA standard curve. For example, a sample with an input of 10 ng into the qPCR would have a QFI of 100% if all 3,030 haploid templates were available for amplification. Similarly, the QFI would be 3.3% if only 100 of the 3,030 input template copies could be amplified to generate the target amplicon.

### DNA quantification using the Qubit® 2.0 Fluorometer

A total of 165 FFPE samples were diluted to 5 ng/μl and quantified using the Qubit dsDNA HS Assay Kit (catalogue number Q32854, Life Technologies) and the Qubit 2.0 Fluorometer (Life Technologies) per the manufacturer’s instruction. Briefly, 1 μl of DNA sample at 5 ng/μl was diluted 200-fold in Qubit dsDNA HS buffer in clear plastic Qubit Assay Tubes (catalogue number Q32856, Life Technologies) and measured on the fluorometer. Prior to taking the measurements, a two-point calibration curve was established using the supplied standards with the kit, at 0 ng/μl and 10 ng/μl. Samples that fell below the limit of quantification of 0.5 ng/ml (0.1 ng/μl, diluted 200-fold) were not reported by Qubit.

### ‘SPUD’ inhibition assay

To evaluate any PCR inhibition, 32 samples that spanned the spectrum of quality as assessed by QFI-PCR were analyzed using the ‘SPUD’ assay, as described by Nolan *et al*. [16] (Additional file 1). This assay is specific for potato (*Solanum tuberosum*) and lacks homology with human DNA. In this assay, PCR inhibition is revealed by a change in the quantification cycle (C_q_) value and is indicative of inhibitors in the sample tested. The PCR cycling conditions were 95°C for 10 minutes, 50 cycles of 95°C for 15 s, and 60°C for 1 minute.

### Titration of FFPE functional copy number and MiSeq sequencing

To investigate the impact of limiting the FFPE DNA input on mutation calling, a dilution series of two well characterized FFPE DNA samples was created. Sample A was a residual clinical colon FFPE sample with a BRAF V600E mutation determined at a NGS read frequency of 30.0 ± 3.4% across 19 independent targeted NGS runs, using a combination of an internally developed PCR-based enrichment panel (SuraSeq™, Asuragen) [14] and the Ion AmpliSeq Cancer Panel and sequenced on the PGM. Sample B was also a colon FFPE tumor resection, but contained a known PIK3CA H1047R variant present at 38.4 ± 4.6%, averaged across six NGS runs using the same methodologies. For both samples A and B, the mutations were sequenced at a median read depth of >3,000× in all runs. Each of these DNA samples revealed a QFI of 9%. DNA from these FFPE samples was used to generate a 2-fold dilution series from 10 ng to 78 pg (3,030 to 24 haploid DNA copies; Additional file 2), and amplified using targeted NGS as described in Hadd *et al*. [14]. Each sample (titration point) was then tagged with a unique barcode and sequenced on the MiSeq (Illumina, San Diego, CA, USA).

### AmpliSeq cancer panel enrichment, PGM sequencing, and Torrent Server plugin variant analysis

Forty-four FFPE samples spanning the spectrum of DNA quality (as determined by the QFI-PCR) were enriched for 190 amplicons using the Ion AmpliSeq Cancer Panel, barcoded, and sequenced on the Ion Torrent PGM, per the supplier’s instructions. Briefly, 10 ng of DNA (as measured by Nanodrop spectrophotometer) was amplified using the supplied reagents for 20 cycles. PCR products were ligated with barcodes and re-amplified for seven cycles, purified, quantified with a BioAnalyzer 2100 (Agilent, Santa Clara, CA, USA) and normalized. Samples were then pooled into 4 tubes, each containing 10 to 12 samples. Each sample was diluted using nuclease free water and 100 million copies were used in the emulsion PCR reaction using Ion OneTouch™ (Life Technologies). Template-positive ion sphere particles were enriched using the Ion OneTouch™ ES system (Life Technologies) and subsequently sequenced on an Ion Torrent PGM following the manufacturer’s recommendation on four 318 chips. Raw sequence data are available for download through the NCBI under the following accession ID: PRJNA212586 [17].

All data analysis was performed using the Torrent Suite v3.2.1 (Life Technologies). Samples were automatically split using pre-defined barcodes and reads were aligned to the reference human genome hg19. Variant calling was performed using the plugin v3.2.4. Over 99% of all variants reported by the plugin had at least 100 reads (median = 920 reads). Less than 1% of all detected variants were reported as indels by the Torrent plugin. As a confirmation strategy, the same set of 44 FFPE samples was also processed using an internally developed 16 gene PCR enrichment panel [14] and sequenced on the PGM. Variant calling on the 16 gene panel was performed as previously described in Hadd *et al*. [14]. All reported variants from the 16 gene panel were covered by at least 200 reads (median = 5,881 reads). No indel variants detected in the primary AmpliSeq assay overlapped with the region sequenced using the 16 gene confirmation panel. As a result, none of these indels could be confirmed.

## Results

### Variability of FFPE DNA quantification across three methods

**Figure 2:**
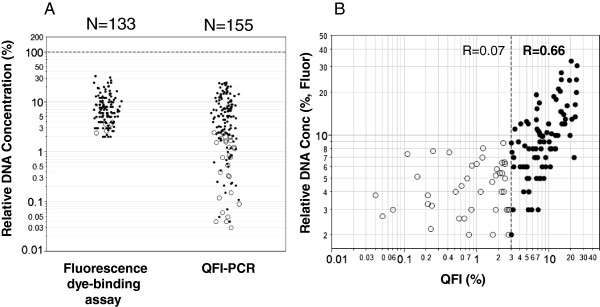
**FFPE DNA characterization by QFI-PCR and fluorescence-based assays from 165 tumor DNA samples. (A)** Distribution of FFPE DNA quantification using QFI-PCR and the fluorescence-based Qubit dsDNA HS assay from 5 ng bulk DNA input as determined by NanoDrop spectrophotometry. A total of 27 samples were undetected by fluorescence assay (<0.1 ng/μl, equivalent to 2% ‘functional’ DNA templates; shown as open circles); these samples produced between 0.03 and 2.5% QFI using qPCR. Five samples were undetected by QFI-PCR and produced between 2.4 to 4.0% of the spectrophotometrically determined DNA concentration using Qubit. Five samples were undetected using both methods (not shown in figure). **(B)** A scatterplot of QFI-PCR and Qubit relative DNA quantification revealed a linear trend for templates with at least 3% QFI (R = 0.66; N = 86). No correlation, however, was observed for the poorest quality samples (R = 0.07; N = 42; shown as open circles).

In our initial studies assessing targeted NGS of tumor FFPE DNA, we observed that DNA quantification varied significantly across spectrophotometric, fluorescent, and qPCR-based assays. This result is clearly shown in Figure 2A, where 5 ng of residual clinical FFPE DNA determined spectrophotometrically (NanoDrop) produced extremely variable results compared to a widely used DNA-binding fluorescent dye assay (Qubit). For example, when three distinct FFPE sample cohorts representing 165 specimens were standardized to 5 ng DNA, the Qubit assay reported a median of 0.30 ± 0.4 ng (interquartile range 0.13 to 0.49). Thus, the NanoDrop reported an approximately 17-fold higher DNA concentration in these FFPE DNA specimens.

In addition to spectrophotometry and Qubit, we also evaluated a qPCR-based assay. Unlike the Qubit assay, qPCR offers an unforgiving and strict requirement for template quality; chemically modified or fragmented DNA strands that cannot be amplified simply drop out during PCR and failed to report a signal, just as they would during PCR-based target enrichment for NGS. To enable a simple, economical, and high throughput assay configuration, we selected a single locus for PCR in the *TBP* gene, chosen because it is devoid of widespread gene amplification or deletion events in cancer. For example, an analysis of copy number changes reported through the cBioPortal for Cancer Genomics revealed an average of 0.8% and 0.4% of samples with deletion or amplification events, respectively, in the *TBP* gene across the 6 cancer types tested in this study [18,19]. Importantly, the amplicon length for this qPCR assay was designed to be 119 bp, or equivalent to the median length of amplicons produced for the associated NGS studies.

The qPCR assay, termed QFI-PCR, was calibrated to high quality cell line DNA (NA04025). QFI-PCR was efficient, sensitive, and repeatable. For example, the PCR amplification efficiency of this assay over four independent runs was 92.6 ± 4.5%. The limit of detection was 5 copies of target, and the limit of quantification was 10 copies (using a criterion of <0.5 standard deviation in C_q_ variability). Thus, the assay can quantify as few as 10 DNA copies, which would correspond to 0.67% amplifiable templates in a background of 5 ng genomic DNA (equivalent to 1,515 haploid copies total input). In repeatability experiments, a mean coefficient of variation (CV) of 26% (95% CI 21.8 to 30.4%) was observed in 3 independent runs with 2 operators, determined across 43 FFPE DNA samples.

For a target FFPE DNA sample, 5 ng of template (nominally determined by NanoDrop) was input into QFI-PCR, and the C_q_ output was converted to a ‘functional’ copy number. The ratio of the calculated sample DNA copy number to the referenced calibrator DNA copy number at the same mass input revealed the fraction of sample templates that were competent for PCR amplification. This fraction, expressed as a percentage, represented the percentage of functional templates, or QFI.

Of the 165 FFPE DNA samples tested using QFI-PCR, 155 (93.9%) yielded a detectable C_q_ value, whereas 10 (6.1%) samples were undetected at 50 cycles. The measured QFI for the detected samples ranged from 0.03 to 24.5%, and the median value was 3.96% (interquartile range 1.22 to 8.55%). A PCR inhibition assay [16], performed on a subset of 32 samples that spanned the range of FFPE DNA quality, showed no signs of inhibition (<1 C_q_ shift), suggesting that samples with low QFI were indeed a consequence of poor DNA integrity (Additional file 1).

In contrast to QFI-PCR, three times as many DNA samples (32, or 19.4%) failed to report a value above the background of the Qubit assay. Moreover, five of these DNA samples were also null in the qPCR assay; these samples represented one-half of the total PCR-negatives. A scatterplot of QFI-PCR and Qubit DNA template functionality (Figure 2B) revealed a linear trend for templates with at least 3% QFI by qPCR (R = 0.66; N = 86). No correlation, however, was observed for the poorest quality samples (<3% QFI). The 79 samples within this group reported a median of 2.6% relative quantification (range 0.0 to 8.8%) using the Qubit assay, but a median QFI of only 0.78% (range 0.0 to 2.86%), suggesting that these samples were highly degraded with little to no amplifiable DNA present.

Similar QFI scores were obtained using a subset of 62 FFPE samples, including at least 7 for each of the 6 cancer types, when the quality was assessed with an alternative gene, *FTH1*, to *TBP*. The average fold change between the two genes was 1.01 (95% CI 0.8 to 1.04), and no samples with a QFI >3% showed more than a two-fold difference (Additional file 3).

### Impact of defined FFPE DNA copy number on NGS variant quantification

We hypothesized that the low QFI associated with many FFPE samples would have an adverse impact on mutation calling in NGS assays. For example, the recommended DNA input into the Ion AmpliSeq™ Cancer Panel - a commercial enrichment method for targeting diagnostically relevant sequences across 46 cancer-associated genes - is only 10 ng. This input corresponds to 3,030 haploid copies of genomic DNA. The number of amplifiable templates in 10 ng of FFPE DNA can readily be calculated from the QFI. For example, the QFI was 1.2% for the 25^th^ percentile FFPE DNA samples with a detectable C_q_, which corresponds to 0.012 × 3,030 = 36 amplifiable templates. If 10 mutant template copies are needed for accurate percentage variant calling by NGS, then one would expect that FFPE specimens with 1.2% QFI would require a minimum of 27.8% (10/36) mutant templates to be reliably quantified. As a result, the risk of false negative results may be elevated in low quality FFPE DNA due to the paucity of amplifiable templates.

To test this hypothesis, we performed a dilution series of FFPE DNA samples for which the oncogene mutations, mutational load, and QFI had been well characterized. Residual colon tumor FFPE specimens with BRAF V600E or PIK3CA H1047R mutations were independently diluted in two-fold decrements. The percentage of mutation was determined for each sample prior to dilution by either 19 or 6 independent targeted NGS runs, with results of 30.0 ± 3.4% BRAF V600E and 38.4 ± 4.6% PIK3CA H1047R, respectively [14]. We selected 10 mutant allele copies as a minimum number required for accurate quantification to minimize stochastic fluctuations in the variant representation. Consequently, we calculated that 33.3 haploid copies (10/0.30) and 26.0 haploid copies (10/0.384) are required to reliably quantify the 30.0% *BRAF* and 38.4% *PIK3CA* mutations, respectively. This copy number requirement, however, increased by more than an order of magnitude when the QFI (9% for both samples) was considered. When this value was factored in, a total of 370 copies (33.3/0.09) of the BRAF V600E sample, and 289 copies (26.0/0.09) of the PIK3CA H1047R sample, were determined to be necessary to accurately quantify the underlying mutations.

**Figure 3:**
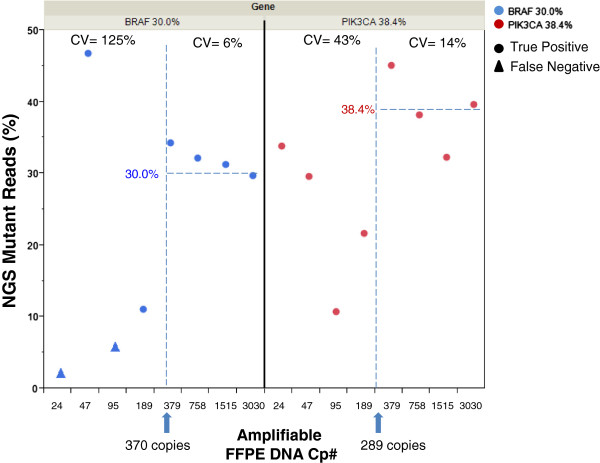
**Effect of amplifiable FFPE DNA copy number on the detection of mutational load by targeted NGS.** The amplifiable DNA copy number (Cp#) for two clinical FFPE samples was calculated to be 370 and 289 copies based on the QFI (9% for both samples) and a well-characterized mutation frequency of 30.0 ± 3.4% for BRAF V600E or 38.4 ± 4.6% for PIK3CA H1047R, respectively. *BRAF* and *PIK3CA* loci were enriched using PCR [14] and sequenced on the MiSeq. The graph shows a dilution series of the DNA, from 24 to 3,030 amplifiable DNA copies and each point (blue, *BRAF*; red, *PIK3CA*) represents the fraction of reads with the target mutation.

The results of the titration series revealed that the detected mutation fraction was highly variable in both samples when the template input was less than 379 copies (Figure 3). For example, at or below 189 template copies, a CV of 125% and 43% for quantification of the *BRAF* and *PIK3CA* mutations, respectively, was observed. This variation stabilized to a CV of 6% and 14% for the same two mutations when the input was increased to 379 copies. The result was in excellent agreement with the theoretical copy number input calculation. Most strikingly, the BRAF V600E mutation was present in <6% of NGS reads when the template was input at 24 and 95 copies. This level of detection is at or below the level of reliable calling from recent published studies of targeted NGS in FFPE DNA [14,20], suggesting a high probability of a false negative.

Nearly 65% of the samples in our 165 specimen cohort had a QFI <6.6%. At this quality threshold, a 10 ng input would correspond to only 200 copies of template. This copy number is approximately the minimum required to accurately quantify a mutation present in 5% of templates when the output is not limited by the NGS read depth. The large fraction of samples in our cohort with this poor quality underscores the importance of understanding the sample-specific DNA characteristics to ensure accurate downstream NGS interpretation. For example, an increase in copy number may be achieved for samples with a borderline QFI relative to variant call sensitivity. If the following formula is used to calculate the bulk DNA input mass into PCR to achieve the reliable quantification of %M mutation:

$$\text{Bulk}\;\text{DNA}\;\text{input}\;\left(\text{ng}\right)=\frac{10}{\left(303*\text{QFI}*\mathrm{\%M}\right)}$$

where the DNA input is expressed in nanograms (bulk DNA mass by spectrophotometry), 303 is the number of haploid copies per 1 ng of DNA, and the QFI is the quantitative functional index, then FFPE DNA inputs may be adjusted accordingly to ensure appropriate library complexity.

### Impact of FFPE DNA quality on NGS false positive calls

Having established the predictive value of a QFI threshold to ensure accurate variant quantification and minimize the risk of false negatives, we next explored the impact of functional DNA quality on targeted NGS using the Ion AmpliSeq Cancer Panel [20]. This panel was selected because of its widespread use, and claims to support FFPE DNA inputs (for example, 10 ng) that may be vulnerable to erroneous mutation calls when low quality FFPE DNA samples are used. Forty-four FFPE DNA samples were enriched across 46 genes and sequenced on the PGM. More than 20 million total reads were generated over four 318 chips with a median of 419,656 reads per sample. The median read depth across all samples was 2,301 and the median C100 (the fraction of bases with at least 100× coverage) was 99.7%. All controls, including FFPE samples that had been previously characterized using an independent NGS method [14], produced the expected results.

**Figure 4:**
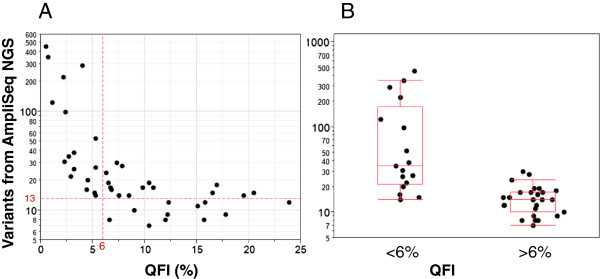
**Effect of FFPE DNA copy number on NGS variant calling using a commercial targeted enrichment cancer panel. A)** Correlation between QFI and number of variants detected on the PGM following enrichment using the AmpliSeq Cancer Panel. **(B)** Samples with the lowest QFI produced a significantly larger number of variants from AmpliSeq NGS compared to those with >6% QFI (*P*-value 0.006).

Analyses of the data revealed an inverse relationship between the QFI and the number of variants detected using the AmpliSeq assay. Samples with a low QFI (that is, functionality of less than 3 to 6%, or about 100 to 200 copies of amplifiable template at 10 ng input) produced a significantly larger number of variants from AmpliSeq NGS compared to those with >6% functionality (*P*-value 0.006, unequal variance) (Figure 4). In fact, >95 variants each were associated with 6 of these low quality DNA samples, of which >75% of the sample-specific variants were detected with a mutation frequency of <10%. A comparison of the mutation frequency among three samples each from the lowest and highest QFI categories is shown in Additional file 4. Since the AmpliSeq Cancer Panel interrogates a 13 kb region of interest, each DNA sample would be expected to present approximately 13 SNPs, based on an incidence of 1 SNP per 1,000 bases [21]. Consistent with this estimate, the mean number of variants for samples with QFI >6% was 14.7 ± 5.8. The mean number of variants for samples with QFI <3%, however, was 166.0 ± 161, suggesting that approximately 90% of the reported variants were false positives.

We next sought to confirm the suspicion of an inflated false positive call rate for the lowest quality FFPE DNA samples. All 44 FFPE samples were analyzed using an internally developed, 16 oncogene PCR enrichment panel that was orthogonal to the AmpliSeq Cancer Panel methodology, sequenced on the PGM, and profiled using a previously described algorithm [14]. Compared to both Sanger sequencing and a high sensitivity liquid bead array method, this targeted NGS method demonstrated excellent concordance in FFPE DNA, and analytical sensitivity to 4% variant [14]. Only those variants from the AmpliSeq Cancer Panel that overlapped with the internal panel content were considered for analysis. Representative samples from each major category of QFI were chosen for this comparison. This set included all 8 samples with QFI of <3%, all 5 samples with QFI between 6 and 7%, and the 6 samples with the highest QFI.

From the primary, unfiltered analysis, only 13% (26/199) of the positives detected by AmpliSeq NGS for samples with <3% QFI could be confirmed (Table 1). At least three categories of false positives were present: i) systematic positives in the *PIK3CA* locus that were present in both high and low quality FFPE samples; ii) low abundance variant calls that were below the threshold of the NGS-based confirmation assay (that is, <4%); and iii) positive calls that were within the range of analytical sensitivity of the confirmation assay (that is, ≥4%). To ensure maximum relevance in the final results, only variants with >5% mean allele frequency from AmpliSeq NGS were evaluated for the calculation of true positives, and systemic variants in the *PIK3CA* gene were excluded. Even after the use of these filters, only 44% of the detected variants could be confirmed for samples with <3% QFI. In contrast, 90% and 96% of variants were confirmed with samples that had 6 to 7% and at least 16.5% QFI, respectively (Additional file 5). KRAS codon 12/13 positives were further confirmed using a second method (liquid bead array) [22], which agreed with the results of confirmation sequencing for the common mutations. As a result, a threshold of 3 to 6% QFI clearly distinguished expected from artificially inflated variant populations and offered a benchmark to support accuracy in variant calling. This threshold is also well matched to *ab initio* calculations of template library complexity relevant to low-level mutation calling in tumor specimens.

**Table 1:** Correlation of FFPE DNA ‘functional’ quality (QFI) with the incidence of false positives in targeted NGS

**Sample**	**Quality by QFI-PCR**	**QFI (%)**	**Percentage functional (Qubit)**	**Total number of mutations from AmpliSeq**	**Overlapping variants in 16 gene panel**	**Overlapping variants in 16 gene panel (PIK3CA false positives removed**^**a**^**)**	**Overlapping variants in 16 gene panel (PIK3CA false positives removed**^**a **^**and >5%)**	**Variant confirmed with independent run (16 gene panel)**	**True positives (%)**	**Number of germline SNPs annotated in dbSNP**	**Confirmed COSMIC mutation**
RS00863	Low	0.5	4	451	48	44	28	3	10.7	2	BRAF V600E 23.8%
RS00856	Low	0.7	5	352	42	36	26	3	11.5	2	KRAS G13C 29.0%
RS01279	Low	1.1	4	123	23	19	8	3	37.5	3	None
RS00865	Low	2.2	4	222	33	30	21	3	14.3	2	NRAS G12R 23.0%
RS01283	Low	2.3	0	31	9	6	3	3	100.0	3	None
RS01282	Low	2.4	5	98	21	17	10	3	30.0	3	None
RS01289	Low	2.7	2	35	11	8	5	4	80.0	3	None
RS01274	Low	2.9	3	22	12	9	6	4	66.7	3	NRAS Q61R 42.6%
RS00866	Medium	6.3	7	24	11	8	5	5	100.0	3	NRAS Q61H 72.1%
											PIK3CA H1047R 12.4%
RS00860	Medium	6.5	7	19	7	4	3	3	100.0	3	None
RS01294	Medium	6.6	8	8	3	3	3	3	100.0	3	None
RS00875	Medium	6.7	5	17	6	3	2	1	50.0	1	None
RS00855	Medium	6.8	11	16	8	5	3	3	100.0	2	KRAS G12D 37.8%
RS00876	High	16.5	12	15	6	4	4	3	75.0	2	PIK3CA H1047R 43.6%
RS01291	High	16.9	13	18	9	6	3	3	100.0	3	None
RS00871	High	17.8	16	9	4	2	2	2	100.0	2	None
RS00873	High	19.5	10	14	6	3	3	3	100.0	3	None
RS00857	High	20.5	13	15	5	4	4	4	100.0	3	KRAS G12V 29.8%
RS00877	High	23.9	20	12	7	4	4	4	100.0	3	KRAS G12S 48.0%

^**a**^To ensure maximum relevance in the final results, only variants with >5% mean allele frequency from AmpliSeq NGS were evaluated for the calculation of true positives, and systemic variants in the *PIK3CA* gene were excluded. True positives were confirmed from AmpliSeq NGS variant calls using an independently designed 16 gene confirmation panel [14].

## Discussion

As NGS technologies advance into clinical settings, it is critical to establish quality control metrics that can guide reliable sequencing results. To this end, entities such as the Next-generation Sequencing Standardization of Clinical Testing (Nex-StoCT) workgroup (coordinated by the Centers for Disease Control), and the College of American Pathologists have proposed criteria for assuring quality NGS data and interpretations. For example, Nex-StoCT recommended a series of post-analytical quality control metrics relevant to NGS, including depth and uniformity of coverage, transition/transversion ratio, base call quality score, mapping quality, and others [23]. Pre-analytical quality control metrics, such as determining the minimum DNA requirements needed to perform the test, are also critical. Although DNA characterization using spectrophotometry is appropriate for many molecular tests and specimen types, FFPE DNA samples pose unique challenges, particularly for amplification-based assays. Targeted detection of FFPE DNA analytes by NGS, a method that offers true digital quantification, demands a careful consideration of template library complexity to achieve reliable and accurate results.

In this study, we compared three assays for assessing FFPE DNA inputs into targeted NGS. The first was spectrophotometry, a method that only reports the ‘bulk’ DNA concentration. Compared to the other quantification methods, this approach overestimated the ‘functional’ DNA concentration by approximately 15-fold across 165 FFPE DNA samples. As a result, we can conclude that spectrophotometry is inappropriate to determine FFPE DNA inputs into PCR-based NGS enrichment since it provides no information to ensure accurate results with both low and high quality DNA samples.

The second comparator method was a fluorescent dye-binding assay (Qubit). This assay is widely used, and offers simplicity, sensitivity, high throughput, and tolerance to various contaminants. Interestingly, we find that the fluorescent dye in this assay is affected by DNA modifications introduced by the fixation/embedding process and thus behaves as a ‘poor man’s’ structural probe that can segregate the highest and lowest quality DNA samples relative to PCR amplification (Figure 2). However, this assay is not capable of differentiating among lower quality FFPE DNA templates (that is, QFI <3 to 6%), nor can it prescribe specific adjustments in DNA input that may help offset the deleterious effects of poor functional quality. In fact, the number of variants called in AmpliSeq NGS was inflated more than 7-fold for the 5 lowest quality FFPE DNA samples in our 44 sample subset when stratified by QFI (median 222 variants) compared to Qubit (median 31 variants). Thus, QFI-PCR, but not Qubit, identified the lowest quality samples at the level of NGS variant calls. Moreover, correlation between QFI-PCR and Qubit by template ‘functionality’ for these samples was poor (R = 0.30). Although a comprehensive understanding of the molecular features of the binding of this particular dye to DNA is lacking, the binding site for similar dyes is only two to four nucleotides [24]. One explanation for this observation is that the lowest quality FFPE samples contain DNA fragments that are receptive to dye binding and fluorescent signal enhancement, but that these samples are poorly amplified by PCR. This is a critical insight that must be accommodated when the downstream assay is based on PCR enrichment, as it is for many targeted NGS assays.

In contrast, we find that the third method, QFI-PCR, is well suited to profile FFPE DNA intended for targeted amplification prior to NGS. First, it is logical to design a quality control with the same methodology that is used for targeted enrichment. For this reason, we designed the target amplification region of QFI-PCR to match the median amplicon size produced by the AmpliSeq Cancer Panel multiplex PCR. Second, QFI-PCR offers absolute quantification that can be used independent of other methods to calculate a minimum copy number input to satisfy downstream assay requirements. Third, the assay is sensitive to PCR inhibitors, and thus can predict potentially poor performance in library enrichment due to extraction contaminants. QFI-PCR respects that high template quality is not the sole sample-level variable that drives successful library preparation. Lastly, the assay is cost-effective, high throughput, and leverages a ubiquitous install base of real-time thermal cyclers that can facilitate adoption by research and clinical laboratories.

It is important to note that the utility of QFI-PCR depends on the use of a genomic locus that is unaffected by tumor ploidy such that the measured copy number in FFPE DNA reflects the functional quality of the DNA specimen and not a separate process. We selected a region in the *TBP* gene since this gene was reported to be unchanged in copy number in >98% of TCGA samples from the cancers investigated in this study. Caution is warranted for use of this locus in neoplasms such as adenoid cystic carcinomas that have a high rate of *TBP* amplification or deletion. In these cases, other genes such as *FTH1* may be targeted instead. Alternatively, a single multiplex assay that includes both *TBP* and *FTH1* loci may be useful.

Importantly, the results of QFI-PCR can be used to calculate the minimum amount of sample input for targeted PCR enrichment by measuring the percentage of DNA templates that are competent for PCR amplification. This insight can reduce the risk of false positives and false negatives in variant calling using both laboratory-developed and commercially available procedures for enrichment and subsequent NGS. This conclusion is evinced in Figures 3 and 4, and Table 1, which demonstrate that: i) false negatives and inaccurate mutation fractions can be rescued by increases in DNA input that are guided by the QFI; and ii) a commonly used commercial method for the multiplexed PCR enrichment of cancer genes can produce an overwhelming number of false positives if the ‘functional’ DNA copy number is unacceptably low. As a result, the integration of a pre-analytical step based on QFI-PCR offers a much improved approach to ensure accuracy in NGS data interpretations. This advance is particularly timely since ‘benchtop’ NGS instrument placements now number in the thousands and many solid tumor tests that are currently performed can (and are) being rapidly supplanted by PCR-based targeted NGS assays.

Our results have important implications not only for the evaluation of FFPE DNA prior to NGS, but also for other assays that rely on PCR amplification. Rigorous and quantitative characterization of DNA-poor samples is essential to ensure that results are generated from sufficient copies of functional DNA templates, interpreted with consideration of DNA quality, and can support reliable mutation calls. The consequences of a misguided diagnostic decision based on sequencing results from inadequate amplification of DNA template are serious and could lead to inappropriate patient treatment by failing to identify an actionable mutation or prescribing the wrong treatment based on a false positive result. Such errors may also undermine retrospective biomarker association studies relevant to cancer drug development. To this point, we observed that 75% of the false positives reported in Table 1 were C>T or G>A transition mutations. Repair of FFPE DNA with uracil DNA glycosylase has recently been reported to reduce the incidence of such artifactual mutations [25,26] and other potentially restorative methods for treating FFPE DNA [27] have been described. These approaches may be particularly beneficial for low quality FFPE DNA, which will require very high analytical sensitivity for variant detection to capture a broader group of clinically relevant mutations, such as those encountered in early stage drug resistance [28,29]. In addition, we note that quantitative sample characterization is required for the accurate determination of the mutational load in a tumor, which may have therapeutic value [30]. It is not surprising, then, that confirmation testing is an indispensable component of existing clinical NGS recommendations to guard the accuracy of variant calls [23]. We suggest that quantitative FFPE sample characterization using threshold-based metrics such as the QFI can improve the cost, efficiency, and accuracy of confirmation, and help de-risk the final clinical report. Although FFPE DNA quality can vary considerably across cohorts, the concerns are most acute with the lowest quality specimens. In this study, the median QFI was less than 7% for each of the three collection cohorts, and across all 6 of the tissue types tested (colon, lung, skin, ovary, breast, and thyroid). In fact, nearly half (79/165, 48%) of the 165 FFPE samples in our cohort had a QFI of <3%.

## Conclusions

We recommend routine, functional characterization of FFPE tumor DNA samples prior to PCR-based targeted NGS, particularly when the goal is low-level mutation detection in heterogeneous cancer samples. Although a correlation was observed in the ‘functional’ DNA fraction comparing QFI-PCR and the fluorescence-based Qubit assay, this correlation was limited to comparatively high quality FFPE samples. That said, the Qubit assay is clearly superior to spectrophotometry for the assessment of template suitability in PCR, and can provide specificity to identify those samples that are most likely to provide high performance NGS results. What this dye-binding assay cannot do, however, is reliably pinpoint the poorest quality samples, or guide changes in procedure that can help offset the negative impact on NGS calls. Without this guidance, many lower quality FFPE DNA samples that may be well accommodated in targeted NGS might otherwise be eliminated for testing. For this reason, we advocate the utility of QFI-PCR, which provides absolute template quantification that is responsive to the minimum library complexity required for accurate NGS results. The actual number of functional copies needed for an NGS assay depends on the target detection sensitivity; for example, if at least 10 ng of DNA is required to detect a 5% mutant in a sample with a 6.6% QFI, then twice as much DNA (and thus twice the template copy number) is needed to detect a 5% mutant in a sample with a 3.3% QFI. By offering a quantitative foundation to define pre-analytical, sample-specific molecular variables, assess template complexity, and inform input corrections that can help guard against erroneous mutation calls, QFI-PCR offers a much needed tool to help meet the challenge of accurate NGS clinical testing of FFPE tumor biopsies.

## Abbreviations

Bp: Base pair; CI: Confidence interval; Cq: Quantification cycle; CV: Coefficient of variation; FFPE: Formalin-fixed paraffin-embedding/embedded; HS: High sensitivity; Nex-StoCT: Next-generation Sequencing Standardization of Clinical Testing; NGS: Next-generation sequencing; PCR: Polymerase chain reaction; PGM: Personal Genome Machine; QFI: Quantitative functional index; qPCR: quantitative polymerase chain reaction; SNP: Single-nucleotide polymorphism.

## Competing interests

SS, LC, JH, JK, ACM, RZ and GJL are employees of Asuragen and own stock or stock options in the company. No other competing interests are declared.

## Authors’ contributions

SS performed the quantification assays, targeted NGS, data analysis, and drafted the manuscript. LC performed quantification assays, targeted NGS, and contributed to data analysis. JK carried out the DNA extractions, performed quantification assays and contributed to data analysis. JH performed targeted NGS and contributed to data analysis. ACM and RZ conducted the primary NGS data analysis. GJL conceived and designed the study, supervised the study, performed data ansalysis, and drafted the manuscript. All authors read and approved the final manuscript.

## Supplementary Material

Additional file 1: Table S1 Assessment of DNA quality and inhibition using QFI-PCR and the ‘SPUD’ qPCR assay. Click here for file

Additional file 2: Table S2 Comparison of observed mutation frequency as a function of template copy number. Click here for file

Additional file 3: Figure S2 Comparison of QFI using amplification loci in *TBP* (119 bp) and *FTH1* (119 bp). Click here for file

Additional file 4: Figure S3 Comparison of AmpliSeq NGS mutation frequencies among samples with the lowest and highest QFI. Click here for file

Additional file 5: Table S3 Confirmed AmpliSeq NGS variants across low, medium, and high QFI FFPE DNA samples. Click here for file
