# Supplementary material for: Functional DNA quantification guides accurate next-generation sequencing mutation detection in formalin-fixed, paraffin-embedded tumor biopsies
Source: Genome Med. 2013 Aug 30;5(8):77. doi: 10.1186/gm481 (PMC3978876; doi:10.1186/gm481)
Supplement: Additional file 1: Table S1 — Assessment of DNA quality and inhibition using QFI-PCR and the ‘SPUD’ qPCR assay. [file gm481-S1.pdf]

Supplemental Table 1: Assessment of DNA quality and inhibition using QFI-PCR and the "SPUD" qPCR assay.

Sample #	Sample ID	QFI (%)	SPUD (C_q)
1	RS00855	6.8	25.4
2	RS00856	0.7	25.5
3	RS00857	20.5	25.4
4	RS00858	4.6	25.5
5	RS00859	8.8	25.5
6	RS00860	6.5	25.4
7	RS00861	8.3	25.3
8	RS00862	11.0	25.5
9	RS00863	0.7	25.3
10	RS00864	0.1	25.0
11	RS00865	2.2	25.3
12	RS00866	6.3	25.5
13	RS00867	2.9	25.4
14	RS00868	10.1	25.5
15	RS00869	6.1	25.4
16	RS00870	10.1	24.9
17	I-3961	0.2	25.6
18	I-3962	0.4	25.3
19	I-3972 (2)	0.6	25.5
20	I-3979	0.9	25.6
21	I-3980	1.5	25.5
22	I-3998 (2)	9.2	25.6
23	I-3999	7.5	25.5
24	I-4000	0.9	25.5
25	AF122	0.2	25.4
26	AF123	3.9	25.3
27	AF124	0.0	25.4
28	AF125	12.1	25.6
29	AF126	3.9	25.4
30	AF127	0.0	25.4
31	AF128	0.3	25.6
32	AF129	0.2	25.5
33	No Template Control-Water	NA	25.5
