# Supplementary material for: Functional DNA quantification guides accurate next-generation sequencing mutation detection in formalin-fixed, paraffin-embedded tumor biopsies
Source: Genome Med. 2013 Aug 30;5(8):77. doi: 10.1186/gm481 (PMC3978876; doi:10.1186/gm481)
Supplement: Additional file 2: Table S2 — Comparison of observed mutation frequency as a function of template copy number. [file gm481-S2.pdf]

Supplemental Table 2: Comparison of observed mutation frequency as a function of template copy number. Two FFPE samples with 9% QFI each were titrated two fold from 10 ng to 78 pg.

Input in ng (Y)	Theoretical Cp# (N=Y*303)	Functional Cp# (FC= N*0.09)	Theoretical Mutant Cp# with 30.0% mutant (MC1=FC*0.30)	Obtained mutation fraction-Sample A (BRAF V600E @ 30.0%)	Theoretical Mutant Cp# with 38.4% mutant (MC2=FC*0.384)	Obtained mutation fraction-Sample B (PIK3CA H1047R @ 38.4%)
10	3030	272.7	81.8	29.7	104.7	39.6
5	1515	136.4	40.9	31.2	52.4	32.3
2.5	758	68.2	20.5	32.1	26.2	38.2
1.25	379	34.1	10.2	34.3	13.1	45.1
0.63	189	17.0	5.1	11.1	6.5	21.6
0.31	95	8.5	2.6	5.8	3.3	10.7
0.16	47	4.3	1.3	46.8	1.6	29.6
0.078	24	2.1	0.6	2.1	0.8	33.8
