# Supplementary material for: Functional DNA quantification guides accurate next-generation sequencing mutation detection in formalin-fixed, paraffin-embedded tumor biopsies
Source: Genome Med. 2013 Aug 30;5(8):77. doi: 10.1186/gm481 (PMC3978876; doi:10.1186/gm481)
Supplement: Additional file 3: Figure S2 — Comparison of QFI using amplification loci in TBP (119 bp) and FTH1 (119 bp). [file gm481-S3.pptx]

## Slide 1
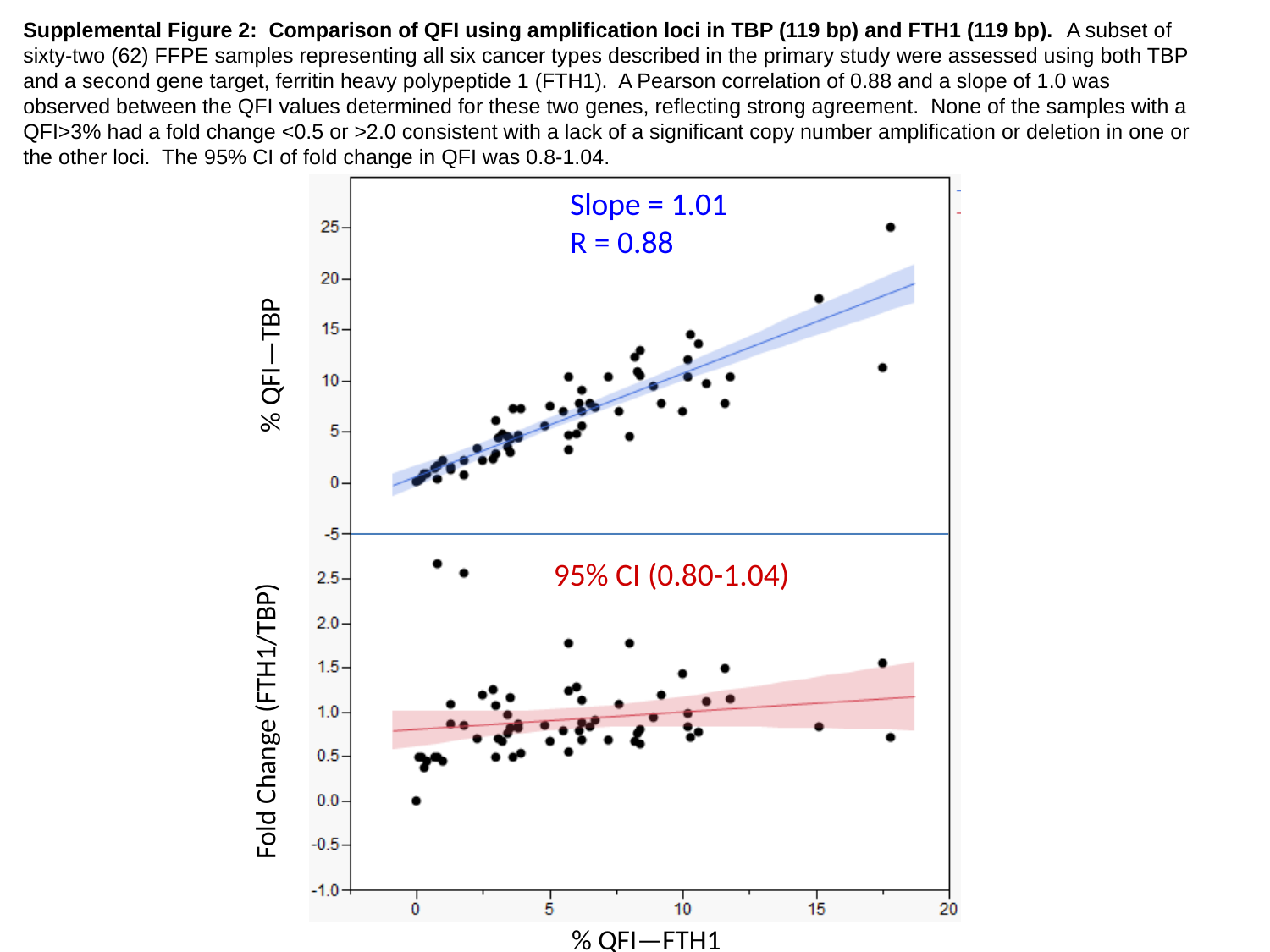

Supplemental Figure 2: Comparison of QFI using amplification loci in TBP (119 bp) and FTH1 (119 bp). A subset of sixty-two (62) FFPE samples representing all six cancer types described in the primary study were assessed using both TBP and a second gene target, ferritin heavy polypeptide 1 (FTH1). A Pearson correlation of 0.88 and a slope of 1.0 was observed between the QFI values determined for these two genes, reflecting strong agreement. None of the samples with a QFI>3% had a fold change <0.5 or >2.0 consistent with a lack of a significant copy number amplification or deletion in one or the other loci. The 95% CI of fold change in QFI was 0.8-1.04.
Slope = 1.01
R = 0.88
% QFI—TBP
95% CI (0.80-1.04)
Fold Change (FTH1/TBP)
% QFI—FTH1
