# Supplementary material for: Functional DNA quantification guides accurate next-generation sequencing mutation detection in formalin-fixed, paraffin-embedded tumor biopsies
Source: Genome Med. 2013 Aug 30;5(8):77. doi: 10.1186/gm481 (PMC3978876; doi:10.1186/gm481)

Supplemental Figure 1: Comparison of the AmpliSeq NGS mutation frequencies among samples with the lowest and highest QFI. The majority of the mutations in poor quality samples have variant frequency <10%. Mutations are colored using AmpliSeq annotation of 739 “HotSpots”.

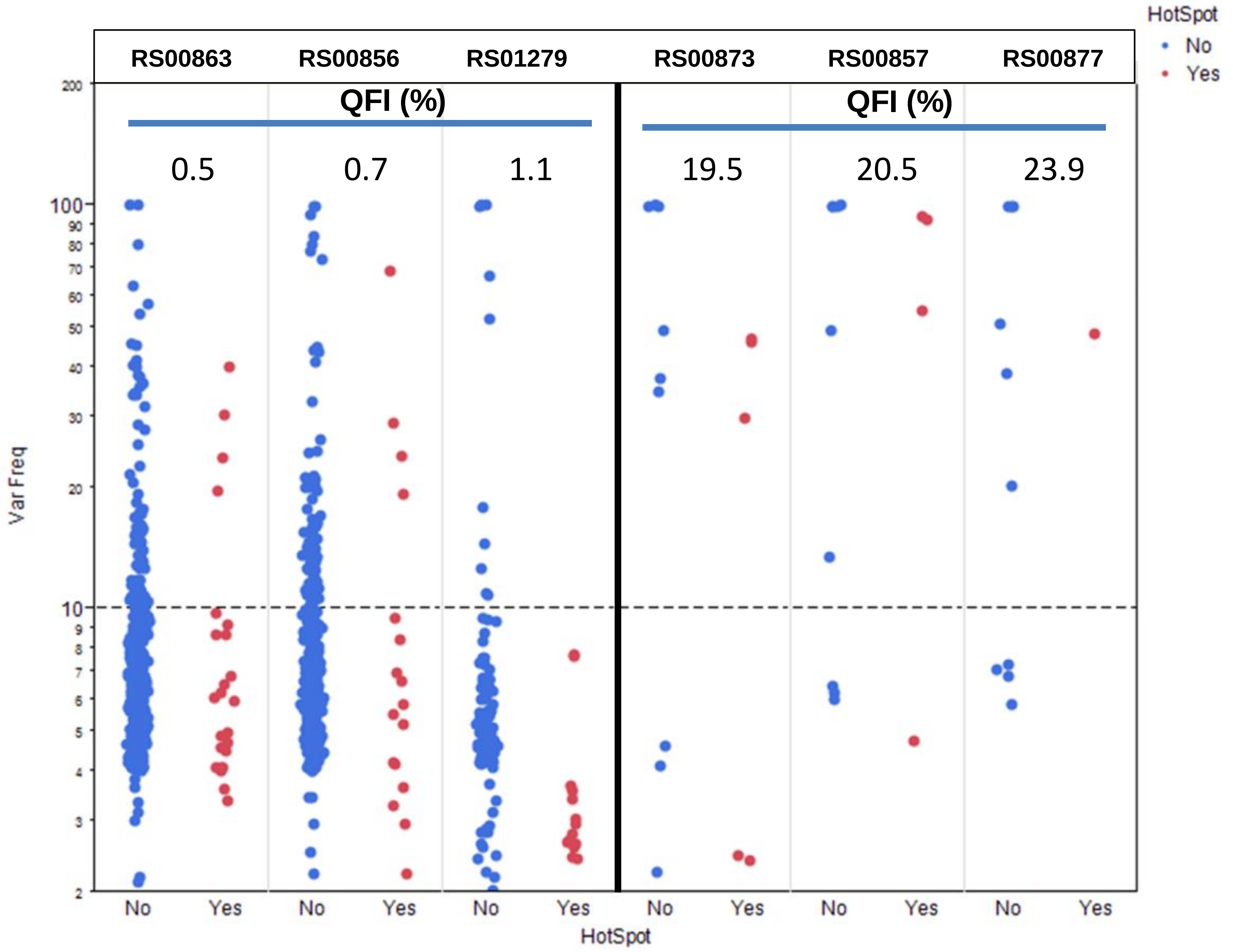

Supplement: Additional file 4: Figure S3 — Comparison of AmpliSeq NGS mutation frequencies among samples with the lowest and highest QFI. [file gm481-S4.pdf]
